# Pharmacogenomic network analysis of the gene-drug interaction landscape underlying drug disposition

**DOI:** 10.1016/j.csbj.2019.11.010

**Published:** 2019-12-05

**Authors:** Yitian Zhou, Volker M. Lauschke

**Affiliations:** a Department of Physiology and Pharmacology, Karolinska Institutet, Stockholm 171 77, Sweden

**Keywords:** Personalized medicine, Precision medicine, Gene-gene interactions, Missing heritability, Drug disposition

## Abstract

In recent decades the identification of pharmacogenomic gene-drug associations has evolved tremendously. Despite this progress, a major fraction of the heritable inter-individual variability remains elusive. Higher-dimensional phenomena, such as gene-gene-drug interactions, in which variability in multiple genes synergizes to precipitate an observable phenotype have been suggested to account at least for part of this missing heritability. However, the identification of such intricate relationships remains difficult partly because of analytical challenges associated with the complexity explosion of the problem. To facilitate the identification of such combinatorial pharmacogenetic associations, we here propose a network analysis strategy. Specifically, we analyzed the landscape of drug metabolizing enzymes and transporters for 100 top selling drugs as well as all compounds with pharmacogenetic germline labels or dosing guidelines. Based on this data, we calculated the posterior probabilities that gene *i* is involved in metabolism, transport or toxicity of a given drug under the condition that another gene *j* is involved for all pharmacogene pairs (*i*, *j*). Interestingly, these analyses revealed significant patterns between individual genes and across pharmacogene families that provide insights into metabolic interactions. To visualize the gene-drug interaction landscape, we use multidimensional scaling to collapse this similarity matrix into a two-dimensional network. We suggest that Euclidian distance between nodes can inform about the likelihood of epistatic interactions and thus might provide a useful tool to reduce the search space and facilitate the identification of combinatorial pharmacogenomic associations.

## Graphical abstract

**Figure:**
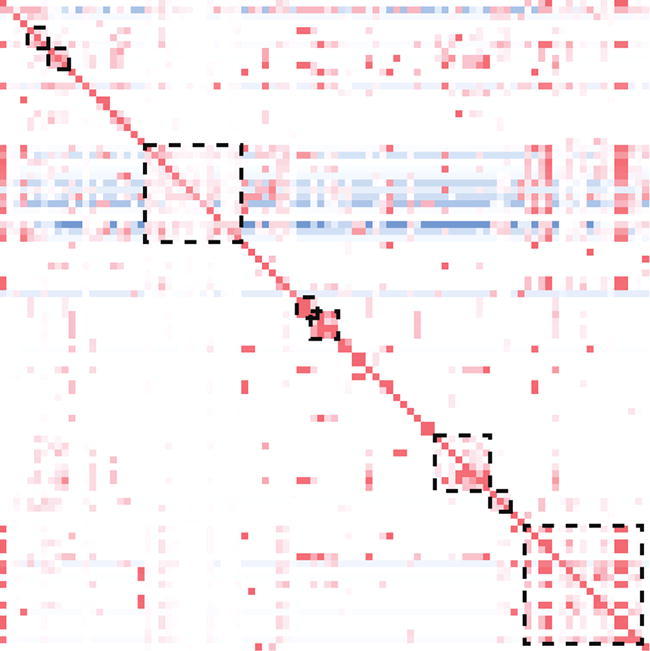

## 1 Introduction

Inter-individual variability in drug disposition is major cause for lack of efficacy or adverse reactions to pharmacological treatment in up to 50% of all patients, posing big challenges for medical care and drug development. Post-market safety events resulting in drug withdrawals, boxed warnings or safety communications affect 32% of all novel therapeutics approved by FDA from 2001 to 2010, leading to substantial economic losses for pharmaceutical industry [1]. Furthermore, epidemiological data from the US shows that adverse drug reactions (ADRs) cause 8.25% and 19.2% increase of hospital stay length and death rate, respectively, and severe ADRs are estimated to be the 4th-6th leading cause of death [2]. It is estimated that 20–30% of these negative effects can be attributed to genetic variations and more than 200 pharmacogenomic biomarkers have by now been incorporated into pharmacogenetic labels that can provide clinically actionable information regarding drug choice or dosing.

The absorption, distribution, metabolism, elimination (ADME) of most drugs are complex and involve multiple enzymes and transporter systems. As a consequence, it is likely that the effects of functional alteration in one ADME protein on drug response phenotypes can be amplified or compensated if they coincide with functional variation in another component involved in the disposition of the same drug [3]. Importantly, while such combinatorial pharmacogenetic effects are plausible, only few examples have been presented to date, including additive effects of functional *CYP2D6* duplications and UGT2B7*2 genotype for codeine toxicity in breastfed neonates [4] and the balance between active *CYP2D6* and *CYP2C19* alleles for amitryptiline toxicity [5]. Importantly, identification of such pharmacogenetic interactions is hampered at least in part by the high complexity of the analytical problem, which poses problems for traditional analysis methods.

Network analysis constitutes a promising strategy within the field of systems pharmacology [6], [7]. Previous seminal work exploited protein-protein-interaction networks as templates to map and predict adverse events due to pharmacodynamic drug-drug interactions using Bayesian networks [8]. Topological community detection and network proximity-based approaches that leverage drug-drug interaction networks and disease modules within the human protein–protein interactome can furthermore facilitate the identification of novel drug-disease associations, which, in turn, can be exploited for rational drug repurposing [9], [10], [11]. In addition, network-based proximity provides a promising measure to identify synergistic drug combinations that increase therapeutic efficacy without additive toxicity [12]. However, network analytical tools have to our knowledge not been applied in the context of pharmacogenomic interactions.

Thus, in order to facilitate the identification of pharmacogenetic interactions, we first systematically analyzed the landscape of drug metabolizing enzymes and transporters for 212 drugs, including the 100 top selling drugs of 2015 as well as all drugs with pharmacogenetic germline labels or dosing guidelines from FDA and pharmacogenetic expert workgroups. Interestingly, these analyses revealed reoccurring patterns of metabolic fates that provide insights into protein-specific overlaps in substrate-specificity. Construction of gene-drug interaction networks based on pharmacological information resulted in the formation of distinct clusters containing chemically dissimilar entities that separated by therapeutic area. Furthermore, mapping of genetic variability data from 60,706 unrelated individuals on this network template reveals areas of highest risk for pharmacogenetic sensitivity.

## 2 Methods and data sets

### 2.1 Drug information

162 drugs with pharmacogenetic labels were obtained from FDA [13], CPIC [14] and DPWG [15]. This list was complemented with 100 top selling drugs, resulting in a total of 212 drugs for analysis. ADME fingerprints of all compounds were extracted from DrugBank.

### 2.2 Pharmacogenetic data

Genetic variability data was obtained from ref. [16]. In short, ADME gene variability was extracted from whole exome sequencing (WES) data from 60,706 unrelated individuals consolidated by the Exome Aggregation Consortium [17] and the functional impact of the identified variants was estimated using a computational prediction framework optimized for pharmacogenetic predictions [18]. The minor allele frequencies (MAFs) of variations classified as functional were aggregated and complemented with the functional variants CYP1A2*1C (rs2069514), CYP1A2*1F (rs762551), CYP2C19*17 (rs12248560), CYP3A4*22 (rs35599367), CYP3A5*3 (rs776746), CYP2B6*22 (rs34223104), CYP2C8*3 (rs10509681, rs11572080), CYP2C9*3 (rs1057910), CYP2E1*2 (rs72559710) and UGT1A1*28 (rs8175347), which were not included in the analyses. Furthermore, rs11572078 in *CYP2C8* and rs2297595 in *DPYD* were removed as false-positive predictions.

### 2.3 Network analysis

Networks were computed and visualized as previously reported [19]. In short, the similarity of all items, both genes and drugs, was calculated based on association strength as $s_{\mathrm{ij}}=\frac{c_{\mathrm{ij}}}{w_{i}*w_{j}}$ with *w_i_* and *w_j_* indicating the total number of occurrences and *c_ij_* denoting the number of co-occurrences of items *i* and *j*. The weighted network used weights of edges assigned based on the number pharmacogenomic labels from FDA, CPIC and DPWG, with gene-drug links without pharmacogenomic labels weighted as 1. The non-weighted network used equal weights for all gene-drug associations irrespective of pharmacogenomic labels. Mapping of the items in a two-dimensional coordinate system is performed by minimizing the objective function $V\left(x_{1},\cdots ,x_{n}\right)=\sum_{i<j}{\|x_{i}-x_{j}\|}^{2}$ with ***x****_i_* = (x_i1_, x_i2_) denoting a vector of the coordinates of item *i* and $\|x_{i}-x_{j}\|$ denoting the Euclidian distance between items ***x****_i_* and ***x****_j_*. Minimization is performed under the constraint $\frac{2}{n(n-1)}\sum_{i<j}\left\|x_{i}-x_{j}\right\|=1$ to avoid the superposition of all points in a single coordinate. This optimization problem was solved using a variant of the SMACOF algorithm for multidimensional scaling [20]. Assortativity was analyzed using the NetworkX package in Python [21]. For clustering we used the sklearn.cluster module in Python. K-means clustering was performed using a maximum of 300 iterations with 0.0001 tolerance. Agglomerative clustering was conducted without distance threshold using ward linkage and Euclidian distances to minimize the variance of the clusters being merged.

## 3 Results

### 3.1 Posterior probability analysis reveals gene- and gene family-specific pharmacological interaction patterns

**Fig. 1:**
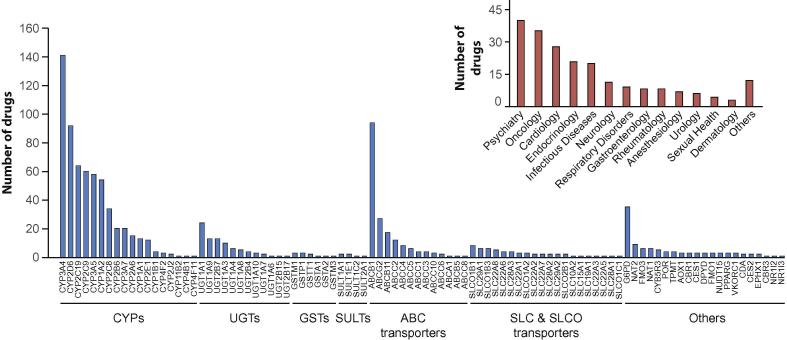
Overview of the genes and drug considered for analysis. Large column plot depicts the number of drug associations per gene. In total n = 212 genes were considered. The inlet shows the distribution of drugs across therapeutic areas.

In total, we extracted ADME information from 212 drugs encompassing 94 associated genes involved in drug disposition or toxicity. The selected drugs were distributed across therapeutic areas with most compounds being used in psychiatry (n = 40), oncology (n = 35) and cardiology (n = 28; Fig. 1). The genes that were implicated in the disposition of most drugs were *CYP3A4* (n = 141 drugs), *ABCB1* (n = 94 drugs), *CYP2D6* (n = 92 drugs) and *CYP2C19* (n = 64 drugs). In contrast, 53 genes were highly specific and only affected 3 drugs or less. Particularly among transporters, nomenclature can differ between the gene and its encoded protein product, as exemplified for *ABCB1* encoding P-gp or MDR1, *SLCO1B1* encoding OATP1B1 and *SLC22A1* encoding OCT1. To facilitate comparisons between gene and protein-related data sets, we consistently use the gene names throughout this work.

To identify patterns across pharmacological signatures, we first calculated the Bayesian posterior probabilities P_i,j_(X_i_|X_j_) for the likelihood that protein *i* is involved in the disposition or toxicity of a given drug under the condition that protein *j* is involved, for all gene pairs (*i*, *j*) with *i* and *j* = 1,…*n*. Based on these values, we derived ΔP_i,j_, defined as the difference between the posterior probability that gene *i* is involved in the disposition of a drug under the condition that gene *j* is involved (P_i,j_(X_i_|X_j_)), compared to the unconditional probability P(X_i_).

**Fig. 2:**
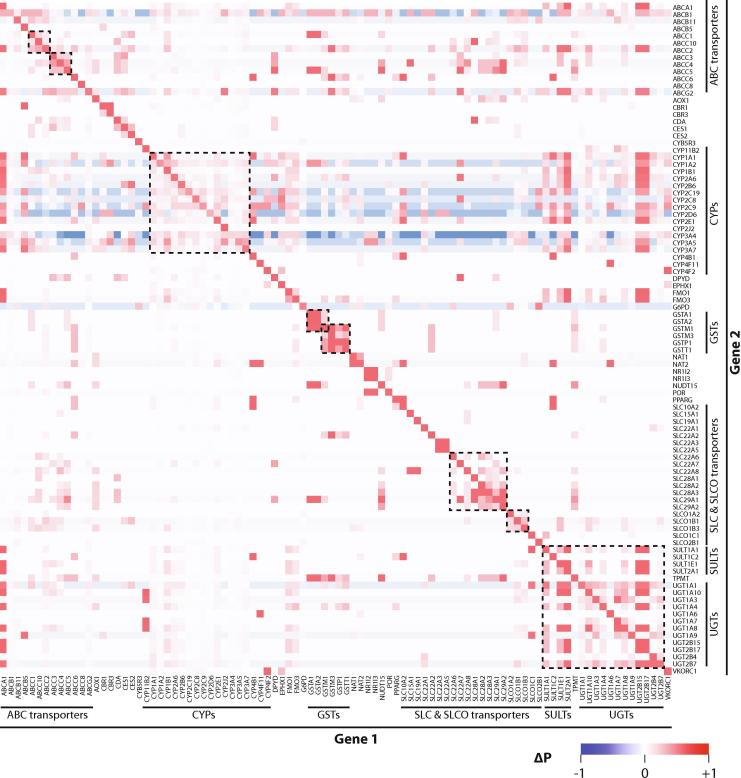
The landscape of metabolic interactions. Heatmap visualization of the difference (ΔP) between the posterior probability that gene 2 is involved in the disposition of a drug under the condition that gene 1 is involved compared to the unconditional probability for metabolism by gene 1, defined as ΔP = P(gene2|gene1) − P(gene2). Blue and red shades indicate combinations for which disposition by gene 1 is a positive or negative predictor respectively. Clusters of metabolic overlap are indicated by dashed boxes. (For interpretation of the references to colour in this figure legend, the reader is referred to the web version of this article.)

We found multiple clusters of functional metabolic overlap (Fig. 2). For instance, GSTA1, GSTA2 and GSTM1 overlapped considerably with ΔP_i,j_ > 0.33. This finding is consistent with studies of azathioprine metabolism for which activities of GSTA1, GSTA2 and GSTM1 were at least one order of magnitude higher than for 11 other GSTs [22]. Interestingly, this pattern is clearly distinct from a neighboring cluster consisting of GSTM1, GSTM3, GSTP1 and GSTT1. Similar phenomena can be observed for the SLCO family of transporters where SLCO1A2, SLCO1B1 and SLCO1B3 share substantial substrate overlap, whereas SLCO1C1 and SLCO2B1 are implicated in the transport of a different set of substrates. Furthermore, we see substantial metabolic interactions among and between members of the UGT and SULT families of phase II enzymes.

CYP metabolism was found to be noticeably correlated, i.e. if a compound is known to be metabolized by one CYP enzyme, the probability that other CYP enzymes partake in the metabolism of the same compound is higher. Furthermore, the likelihood of CYP metabolism is increased if SULT enzymes are involved in drug disposition, whereas, conversely, CYP metabolism is not a good predictor for SULT metabolism. Notably, the identified patterns of metabolic overlap were similar when all drugs with pharmacokinetic information from PharmGKB were considered instead (Supplementary Fig. 1).

**Fig. 3:**
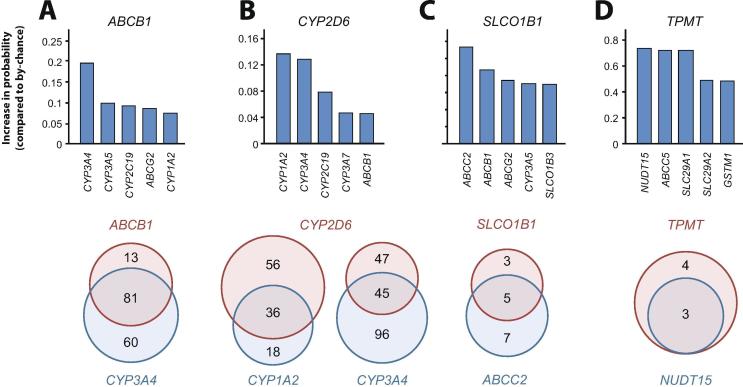
Examples of specific interactions between pharmacogenes. The difference (ΔP) between the posterior probability that gene 2 is involved in the disposition of a drug under the condition that gene 1 is involved compared to the unconditional probability for metabolism by gene 1, defined as ΔP = P(gene2|gene1) − P(gene2) is shown for gene1 as ABCB1 (A), CYP2D6 (B), SLCO1B1 (C) and TPMT (D) and the respective most closely correlated genes. Venn diagrams show the overlap of the number of drugs for the top hits for each respective gene.

In addition to gene family- or subfamily-wide interactions, we found highly specific interactions between individual gene pairs. For instance, of 94 ABCB1 substrates in our data set, 81 were also substrates of CYP3A4 (Fig. 3A). Accordingly, drugs that were transported by ABCB1 were 19.7% more likely to be also CYP3A4 substrates than expected by chance. Similarly, implication of CYP2D6 was a decent predictor of CYP1A2 and CYP3A4 metabolism with ΔP values of 0.14 and 0.13, respectively (Fig. 3B). Notably however, ΔP_CYP3A4,CYP2D6_ substantially lower than ΔP_CYP3A4,ABCB1_. Further notable associations were found for SLCO1B1 that shared substantial overlap with ABCC2 (ΔP = 0.57) and ABCB1 (ΔP = 0.43; Fig. 3C). Finally, TPMT overlapped with NUDT15 (ΔP = 0.74), ABCC5 (ΔP = 0.72) and SLC29A1 (ΔP = 0.72) but not with CYPs (ΔP < 0), UGTs (ΔP < 0) or SULTs (ΔP < 0; Fig. 3D).

### 3.2 Pharmacogenetic network analysis suggests hotspots of pharmacogenetic interactions

**Fig. 4:**
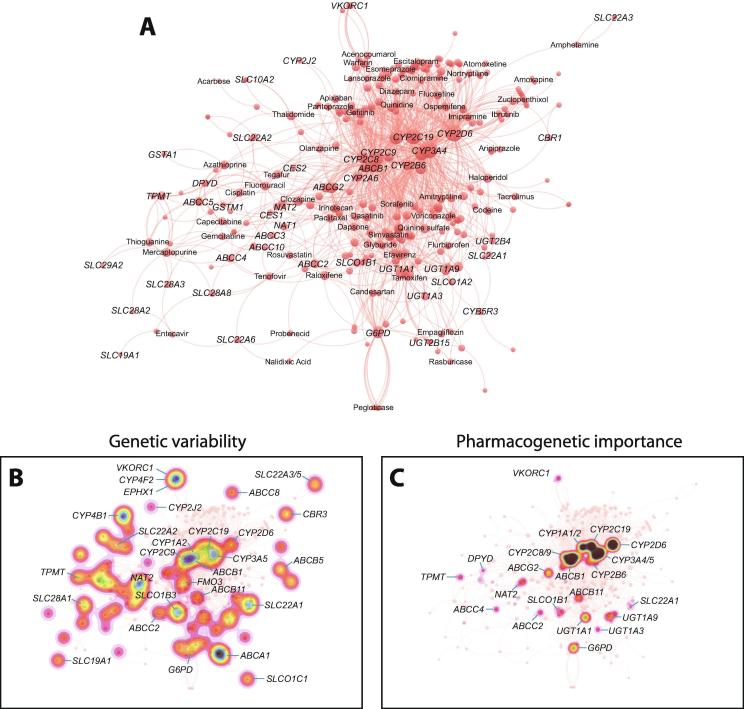
Network analysis of the gene-drug interaction landscape. (A) Network visualization of the pharmacogenetic and metabolic associations across all genes and drugs. Distances between nodes is a measure for node similarity. (B) Using the network in panel A as a template, the genetic variability of the respective pharmacogenes was overlaid (see methods), revealing hotspots of potential pharmacogenetic interactions. (C) Similar as B but considering the number of interactions as measure of pharmacogenetic importance.

To get further insights into the patterns and similarities of metabolic signatures across medications, we used network analytical tools to systematically profile the gene-drug interaction landscape (Fig. 4A). We first generated the network by mapping all analyzed genes and drugs in a two-dimensional coordinate system so that the distance between the nodes constitutes a measure of similarity, whereas node size corresponds to the number of interactions. Network topology was highly similar, irrespective of whether a weighted or a non-weighted mapping approach was used (compared Fig. 4A and Supplementary Fig. 2; see methods section). The resulting network is assortative in nature with an assortativity index of 0.33. This means that pleiotropic ADME genes that metabolize or transport many different medicines cluster preferentially with other pleiotropic ADME genes, whereas ADME genes that associate with only few drugs tend to associate with other ADME genes that also metabolize or transport only few drugs.

To further analyze network structure, we used agglomerative and k-means clustering. Notably, these analyses result in the formation of distinct metabolic clusters that are consistent with our posterior probability analysis, such as clusters of major CYPs, SLCs or UGTs. Interestingly, drugs clustered mostly by therapeutic areas (Supplementary Figs. 3 and 4). Most antidepressants and anxiolytics, including escitalopram, fluoxetine, clomipramine and diazepam clustered closely together indicating similar metabolic fingerprints, whereas antipsychotics, such as clozapine, olanzapine, aripiprazole and haloperidol were clearly separated. ADME patterns alone were moreover sufficient to cluster antineoplastic medications, such as fluoropyrimidine (capecitabine, fluorouracil, tegafur) and thiopurine (mercaptopurine, azathioprine and thioguanine) compounds, as well as cisplatin. TPMT, DPYD, GSTs, as well as ABC and SLC transporters constitute the major metabolic focal points behind this cluster. In contrast, taxanes (paclitaxel) and camptothecin derivates (irinotecan) show different signatures.

Next, we explored how these drug interactions might relate to patterns of pharmacogenetic variability. To this end, we mapped the genetic variability information that we previously extracted from more than 60,000 individuals [16] using the weighted gene-drug interaction network as template (Fig. 4B). We hypothesized that if two genes are highly similar in their metabolic patterns, i.e. are located close to each other in the network, their genetic variability is most likely to result in combinatorial effects. Hotspots of pharmacogenetic variability can be found around *CYP2C8*/*CYP2C9*/*ABCB1*, *VKORC1*/*CYP4F2*/*EPHX1* as well as *ABCA1*/*UGT2B15*/*UGT2B17*/*SULT2A1*. However, once we factored in the number of connections for a given gene as a metric for pharmacogenetic importance, the largest signals can be found around the central cluster comprising CYP genes and *ABCB1*. Further genes involved in the metabolism of multiple clinically relevant drugs with considerable genetically encoded functional variability include *ABCG2*, *UGT1A1*, *G6PD*, *TPMT*, *DPYD*, *SLC22A1* and *NAT2*. Combined, these analyses provide a novel approach to leverage pharmacological interaction data in order to reduce complexity in a combinatorial pharmacogenomics framework, thereby pinpointing potential priority targets for the analysis of gene-gene-drug interactions.

## 4 Discussion

Drug transport and metabolism of many drugs is controlled by genetic factors. Seminal twin studies demonstrated significantly higher intrapair correlations of pharmacokinetic parameters in monozygotic twins compared to dizygotic twins for most evaluated drugs in the published literature, including antipyrine, dicoumarol, nortriptyline, tolbutamide, metoprolol and torsemide with heritability estimates between 80% and 99% [23]. Importantly however, common polymorphisms in genes involved in drug disposition can only account for a minority of the observed variability [24]. Multiple factors have been proposed to contribute to this missing heritability, including rare variants that are not commonly interrogated in pharmacogenomic studies and low power to detect gene–gene interactions [25].

Indeed, rare single nucleotide variants (SNVs) and copy number variations (CNVs) have recently been shown to be highly prevalent in multiple classes of ADME genes, including phase 1 and phase 2 enzymes, as well as various drug transporters [26], [27], [28], [29], [30], [31], [32], [33], and careful estimates suggest that such rare variants might account for up to 20–40% of the functional variability in pharmacogenes [34]. These estimates are corroborated by structural mapping approaches, showing that rare variants can be found in functionally important residues in CYPs [35] as well as SLC [33] and SLCO [32] transporters. Thus, structural evaluations constitute important tools to improve our understanding of functional consequences of pharmacogenetic variants. However, whether rare variant profiling can provide clinically actionable information that can improve patient outcomes remains to be determined [36], [37].

Besides rare variations, gene–gene interactions are suggested to contribute to the unexplained genetically encoded variability in drug disposition. We hypothesized that functional similarities between genes, as defined by shared pharmacological pathways, might flag genes that are more likely to have epistatic interactions. To comprehensively map the gene-drug interaction landscape we employed a network analysis strategy and multidimensional scaling. Interestingly, pharmacological information alone was sufficient to recapitulate structural similarities between drug binding sites. For instance, ABCB1 clustered together with various CYP genes including CYP3A4, whereas other ABC transporters cluster distinctly different. CYP3A4 and P-gp (encoded by *ABCB1*) have been shown to have flexible promiscuous binding pockets [38], [39], resulting in substantial overlap between CYP3A and P-gp substrates and inhibitors [40].

While UGT enzymes cluster closely together in the network, a separation between UGT1 and UGT2 family members can be observed. These findings align with previous functional analyses showing that members of the UGT1 and UGT2 subfamilies have overall overlapping substrate specificity that can differ however in their ability to glucuronidate specific chemical structures, such as tertiary amines or planar phenols [41]. Similarly, network analysis revealed clear functional similarities between the genetically unrelated cation-linked concentrative nucleoside transporters of the *SLC28* subfamily and the *SLC29* family of energy-independent, equilibrative nucleoside transporters [42].

Besides indicating overlaps in substrate specificity, networks can give insights into metabolic interdependencies. For instance, the posterior probability of metabolism by CYPs was much higher if SULTs were involved in the disposition of the respective drug. However, the probability that a compound is metabolized by SULTs was not detectably increased among CYP substrates compared to all drugs. Moreover, GST metabolism and SLC transport with the exception of SLC22A1 (OCT1) and SLC10A2 (ASBT) were largely uncorrelated with CYP metabolism. Notably, community identification in networks constitutes a prolific field of research and a variety of conceptually different algorithms have been presented that rely on centrality measures [43], multifractal or differential network geometry [44], [45], graph modularity [46] or label propagation [47]. Application of these tools to pharmacological and pharmacogenetic networks is an interesting frontier of network research that promises to provide further insights into network function, organization and robustness.

We suggest that pharmacological networks might provide a useful tool for the identification of combinatorial pharmacogenomic effects. Specifically, we argue that genetically encoded functional variability in genes with substantial pharmacological overlap might be more likely to result in complex gene-gene-drug interactions than unrelated genes whose nodes in the network are distant. As such, mapping of genetic variability on the network template, reveals hotspots in which multiple variable genes share functional similarities and might thus represent appealing candidates for the identification of combinatorial genetic effects. From a structural perspective, the high degree of assortativity suggests that the network is rather robust to perturbations, i.e. that disruption of central nodes due to loss-of-function polymorphisms or chemical inhibition is not sufficient to cause the cause the network as a whole to become disconnected. This finding is consistent with the observation that the most severe ADRs, such as fluoropyrimidine toxicity in individuals with reduced DPYD function and mercaptopurine myelosupression in TPMT deficiency, affect nodes with low connectivity. In contrast, disruption of highly connected nodes, such as *CYP2C19* and *CYP2D6*, is common but only rarely results in severe ADRs.

While we believe that our approach constitutes a relevant complement to current analysis methods, multiple limitations have to be considered. The selection of ADME proteins might be biased by the analytical assays, as these are often conducted in batteries. For instance, when investigators are interested in the phase 2 metabolism of a compound of interest, they are more likely to test multiple phase 2 enzymes in parallel instead of testing one isolated enzyme, thus increasing the likelihood to identify substrate overlap. Furthermore, our analysis was based on qualitative pharmacological information and we envision that the integration of quantitative data will further refine interaction networks and facilitate the identification of gene-gene-drug interactions.

In summary, network analyses of gene-drug interactions based on pharmacological information alone resulted in the formation of distinct clusters that can inform about the likelihood of epistatic interactions between pharmacogenes and thus might provide a useful tool to handle the complexity explosion of higher-dimensional interactions, which overwhelms conventional analysis methods.

## Declaration of Competing Interest

V.M.L is co-founder and owner of HepaPredict AB. Y.Z. has no conflicts of interest to declare.
