# Supplementary figures and images for: Pharmacogenomic network analysis of the gene-drug interaction landscape underlying drug disposition
Source: Comput Struct Biotechnol J. 2019 Dec 5;18:52–8. doi: 10.1016/j.csbj.2019.11.010 (PMC6921140; doi:10.1016/j.csbj.2019.11.010)

## Supplementary Figure 1

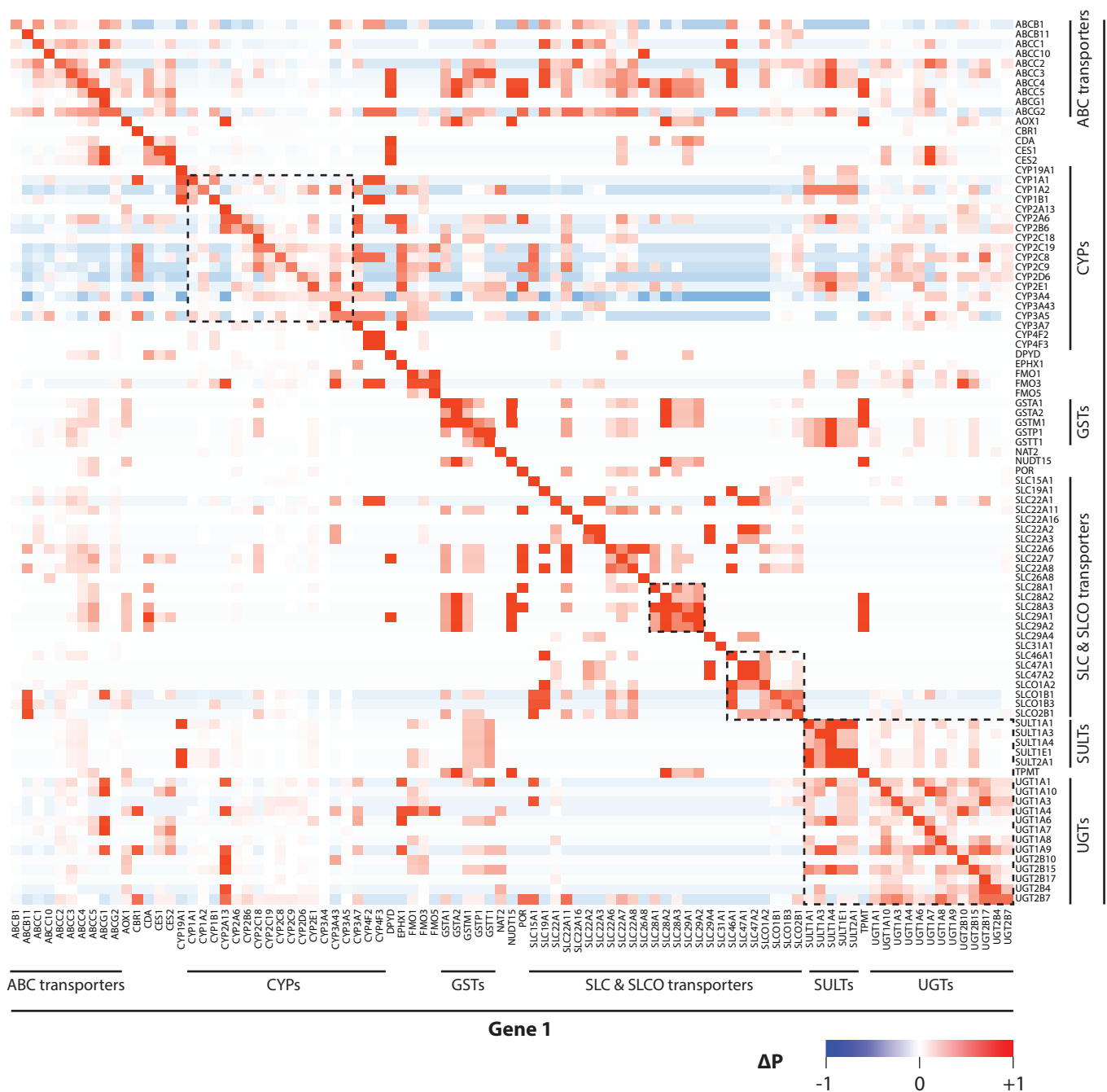

Supplement: Supplementary data 1 [file mmc1.pdf]

## Supplementary Figure 2

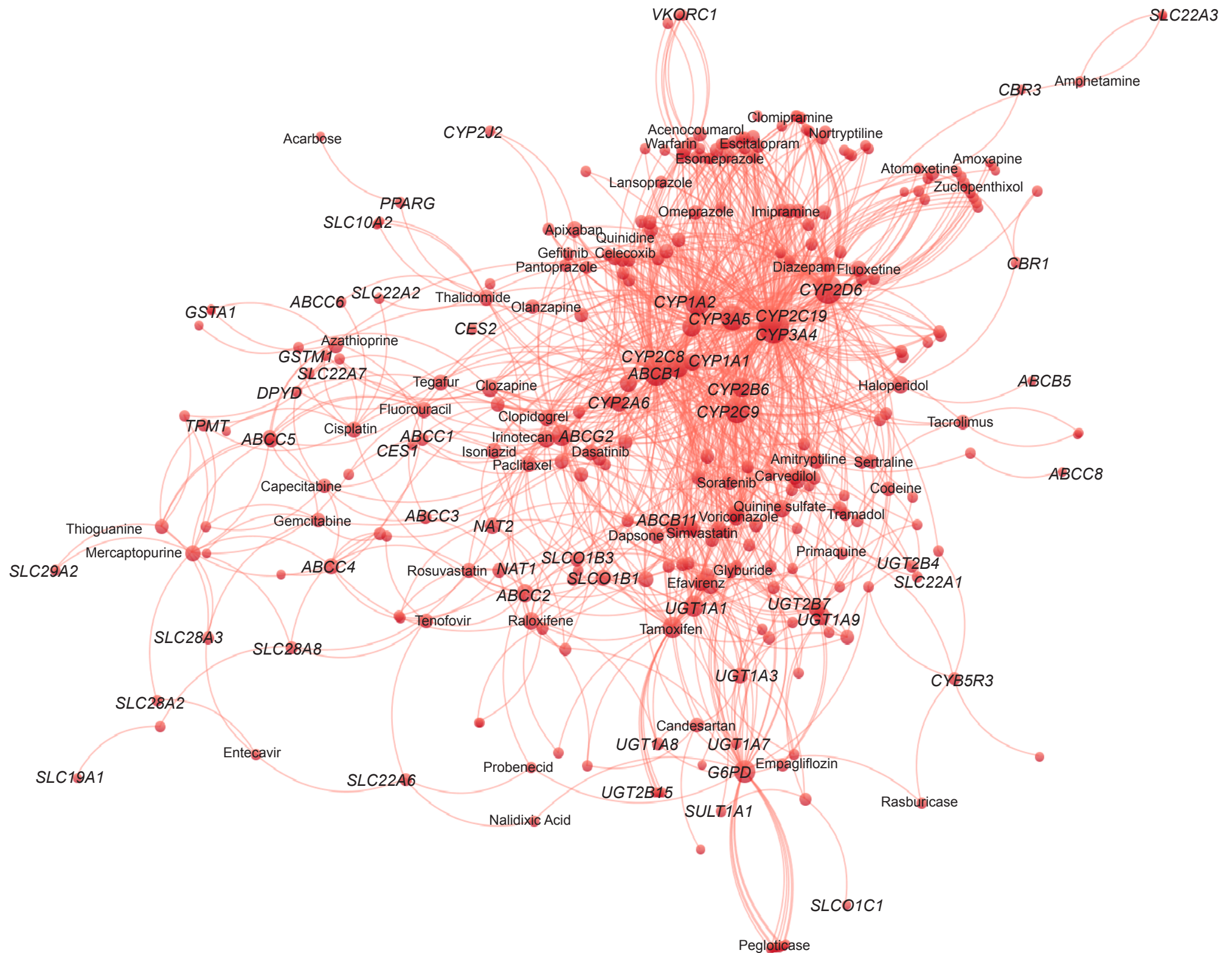

Supplement: Supplementary data 2 [file mmc2.pdf]

# Supplementary Figure 4

A

K-Means Clustering

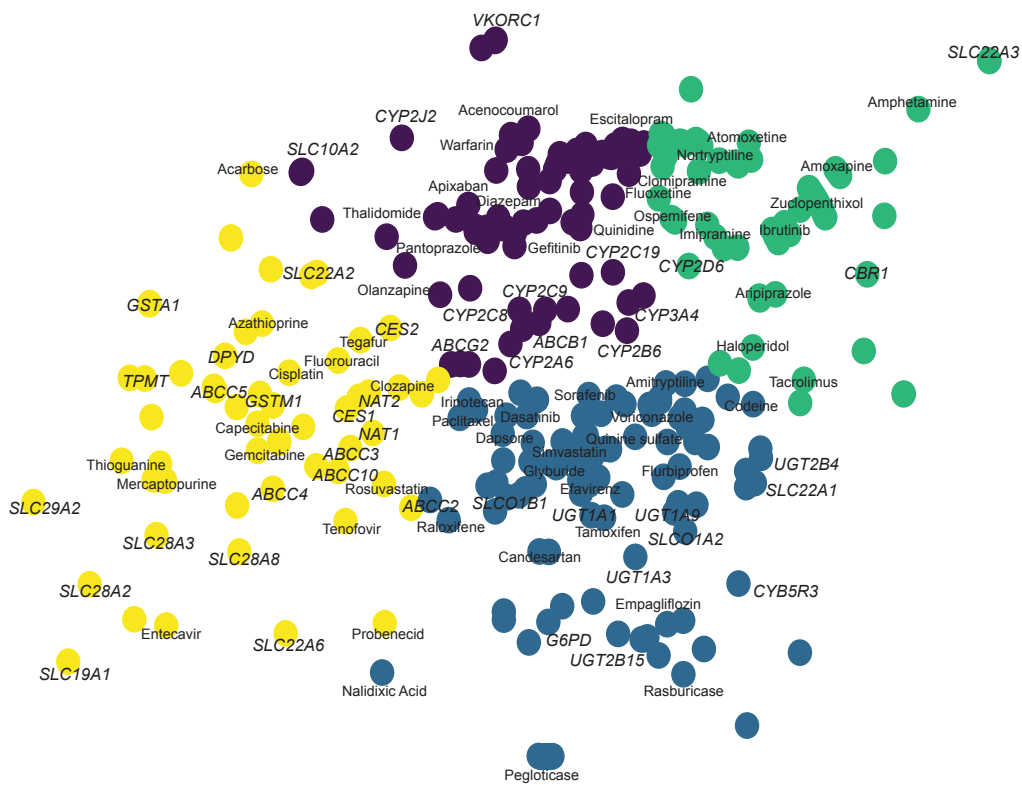

B

Number of drugs in different clusters

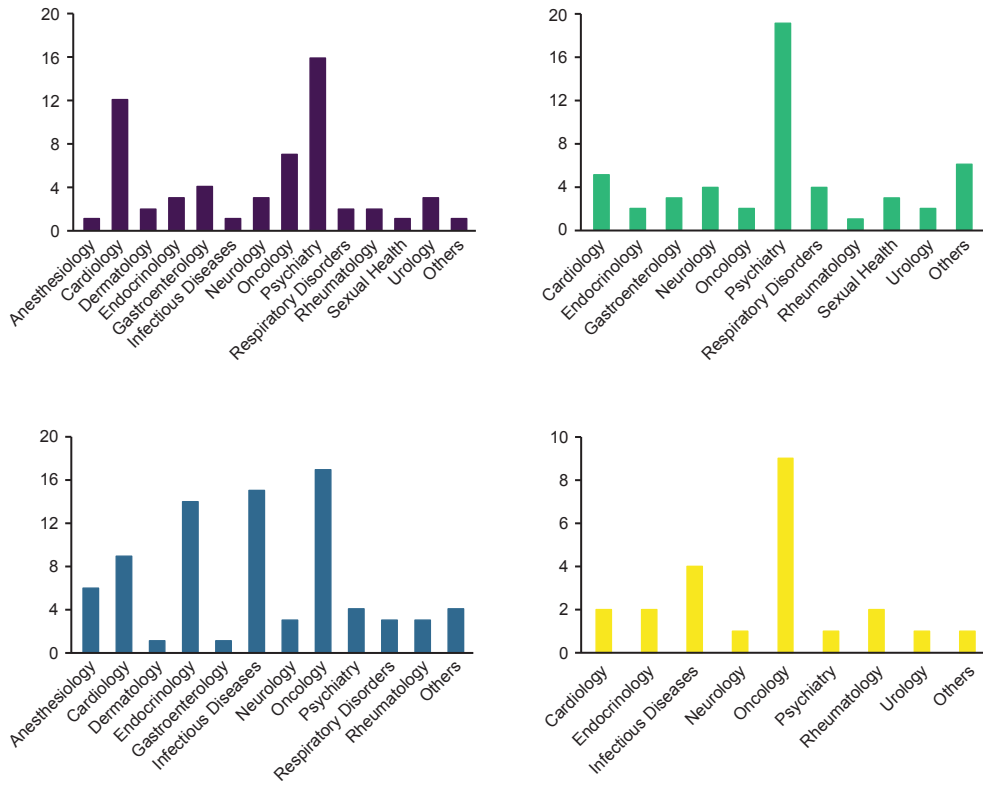

Supplement: Supplementary data 4 [file mmc4.pdf]
