# Supplementary material for: Pharmacogenomic network analysis of the gene-drug interaction landscape underlying drug disposition
Source: Comput Struct Biotechnol J. 2019 Dec 5;18:52–8. doi: 10.1016/j.csbj.2019.11.010 (PMC6921140; doi:10.1016/j.csbj.2019.11.010)

Supplementary Figure 3

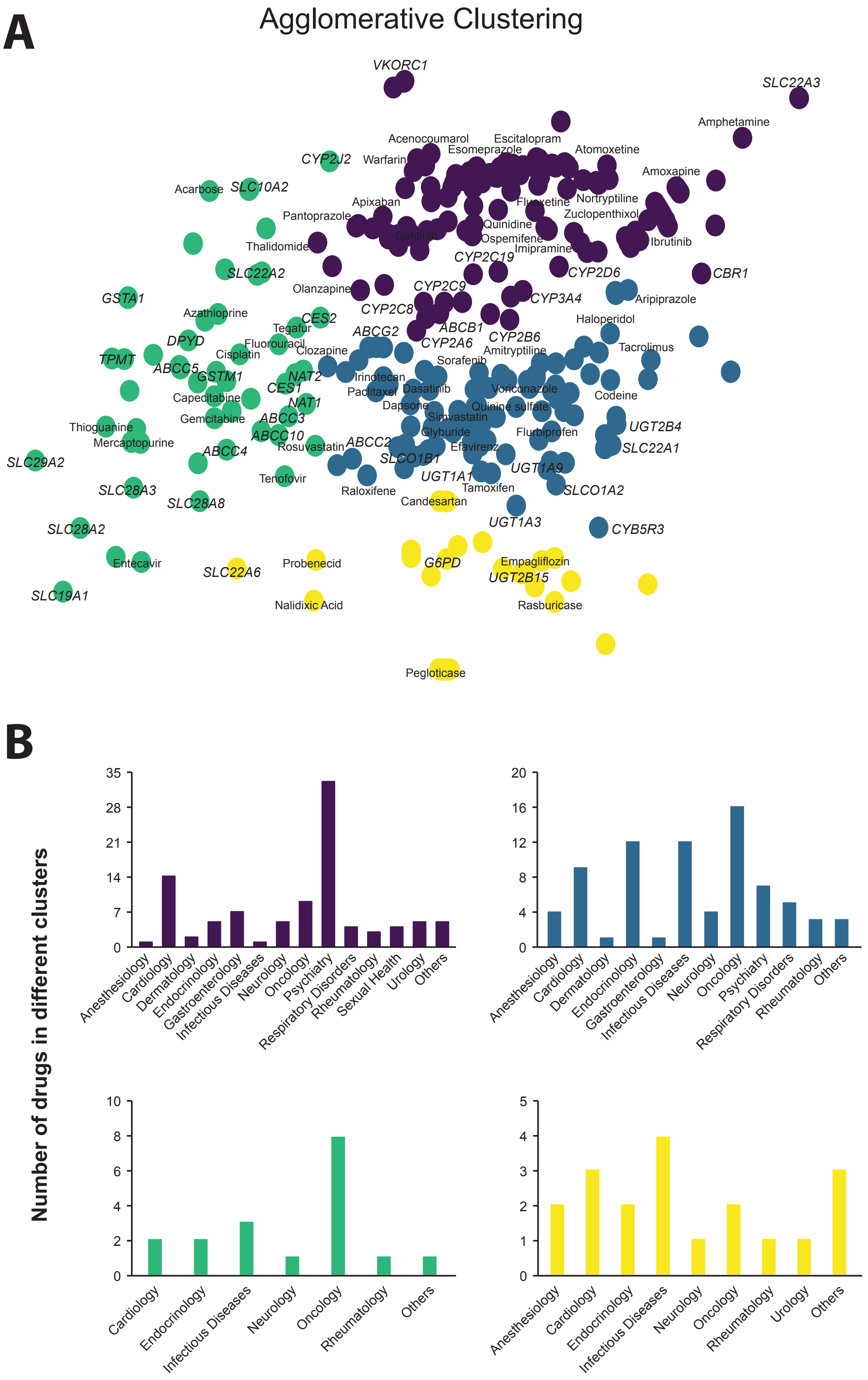

Supplement: Supplementary data 3 [file mmc3.pdf]
